# Overexpression of Ferredoxin, *PETF*, Enhances Tolerance to Heat Stress in *Chlamydomonas reinhardtii*

**DOI:** 10.3390/ijms141020913

**Published:** 2013-10-17

**Authors:** Yi-Hsien Lin, Kui-You Pan, Ching-Hui Hung, Hsiang-En Huang, Ching-Lian Chen, Teng-Yung Feng, Li-Fen Huang

**Affiliations:** 1 Department of Plant Medicine, National Pingtung University of Science and Technology, Pingtung 912, Taiwan; E-Mail: yhlin@mail.npust.edu.tw; 2 Graduate School of Biotechnology and Bioengineering, Yuan Ze University, Taoyuan 320, Taiwan; E-Mail: pku07216@gmail.com; 3 Institute of Plant and Microbial Biology, Academia Sinica, Taipei 115, Taiwan; E-Mails: u100030056@cmu.edu.tw (C.-H.H.); ai0926@hotmail.com (C.-L.C.); natfarm@sinica.edu.tw (T.-Y.F.); 4 Department of Life Science, National Taitung University, Taitung 684, Taiwan; E-Mail: hhn@nttu.edu.tw

**Keywords:** *Chlamydomonas reinhardtii*, ferredoxin, *PETF*, heat tolerance, ascorbate

## Abstract

Reactive oxygen species (ROS) produced by plants in adverse environments can cause damage to organelles and trigger cell death. Removal of excess ROS can be achieved through the ascorbate scavenger pathway to prevent plant cell death. The amount of this scavenger can be regulated by ferredoxin (FDX). Chloroplastic FDXs are electron transfer proteins that perform in distributing photosynthetic reducing power. In this study, we demonstrate that overexpression of the endogenous photosynthetic FDX gene, *PETF*, in *Chlamydomonas reinhardtii* could raise the level of reduced ascorbate and diminish H_2_O_2_ levels under normal growth conditions. Furthermore, the overexpressing *PETF* transgenic *Chlamydomonas* lines produced low levels of H_2_O_2_ and exhibited protective effects that were observed through decreased chlorophyll degradation and increased cell survival under heat-stress conditions. The findings of this study suggest that overexpression of *PETF* can increase the efficiency of ROS scavenging in chloroplasts to confer heat tolerance. The roles of PETF in the downregulation of the ROS level offer a method for potentially improving the tolerance of crops against heat stress.

## 1. Introduction

Excessive greenhouse gas emissions from human activities raise the global temperature, and high ambient temperatures lead to biochemical and physiological changes in plants, thereby affecting plant growth and development. High temperatures cause protein denaturation and aggregation, inhibiting protein function and compromising membrane integrity. Reactive oxygen species (ROS) are subsequently generated when high-energy state electrons are released from heat-disrupted membrane-associated processes such as photosynthesis [1,2]. ROS are highly reactive and toxic, and they can cause oxidative damage to cells [3,4]. To counter the threat of oxidative damage under various environmental stresses, plants have developed ROS-scavenging mechanisms to eliminate ROS [5,6]. By combining antioxidant enzymes with antioxidants, plant cells can detoxify hydrogen peroxide and superoxide [7,8]. Several pieces of evidence indicate that antioxidant enzymes and antioxidants are associated with the plant heat tolerance [7,9–12].

Ferredoxins (FDXs) in chloroplasts are electron transfer proteins that deliver reducing equivalents from photosystem I (PSI) in photosynthetic organisms [13]. Electrons from reduced FDXs are accepted by FDX-NADPH-oxidoreductase (FNR) to generate NADPH, which is required for carbon assimilation in the Calvin cycle [14,15]. FDXs can also donate electrons to nitrite reductase (NiR), sulfite reductase (SiR) and fatty acid desaturases (FADs) for nitrogen and sulfur assimilation as well as fatty acid desaturation [16,17]. In addition, FDXs are key regulators of FDX-thioredoxin reductase (FTR) in thioredoxin systems [14]. Moreover, FDXs are components in the water-water cycle, a ROS-scavenging pathway, and generate ascorbate and peroxiredoxin to protect the photosynthetic apparatus [18–20].

FDX transcripts have been observed to decrease under drought, cold, or salt stress conditions in *Arabidopsis* [21]. The amount of FDX is also decreased in tobacco under various stresses [22]. Decreasing FDX by antisense RNA in transgenic plants causes leaf yellowing under high light stress, and the ROS level is increased in FDXs-limiting plants [23,24]. These results suggest that the expression of FDXs is down regulated by abiotic stress, resulting in increased the ROS level and subsequent oxidative damage to cells. In addition, ectopic expression of a cyanobacterial flavodoxin, which is a functional analog of FDXs found in cyanobacteria and some algae, decreases the ROS level in transgenic tobacco and enhances plant tolerance to heat, high light, chilling, drought, UV radiation, and iron starvation [22,25,26]. However, ectopic expression of a cyanobacterial FDX in tobacco chloroplasts does not improve the tolerance of transgenic plants to oxidative and chilling stresses [27]. Although the level of foreign cyanobacterial FDX has been shown to decrease in the manner of an endogenous FDX in transgenic tobacco under stress [27], whether FDX functions under adverse environment stresses remains uncertain.

The single-celled green alga *Chlamydomonas reinhardtii* is an excellent photosynthetic model organism for examining physiological responses of cells under abiotic stresses [28]. Recent studies on hydrogen production by FDXs and hydrogenase in *Chlamydomonas* have proposed methods for potentially generating clean energy [29–31]. Previous studies have shown that *Chlamydomonas* contains six FDXs, PETF, and FDX2—FDX6 [32,33]. Although the expression levels of *PETF*, and *FDX2*–*FDX6* vary under hypoxia, iron- and copper-deficient conditions [34], PETF is a major photosynthetic ferredoxin in chloroplasts and performs a function in electron transfers between PSI and FNR [34,35]. In this study, we generated transgenic *Chlamydomonas* overexpressing *PETF* to clarify whether increasing FDX gene expression levels enhance the tolerance of algae to heat stress.

## 2. Results

### 2.1. Generation and Characterizations of Transgenic Lines Overexpressing

**Figure 1:**
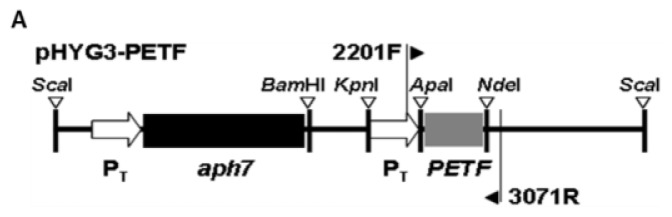

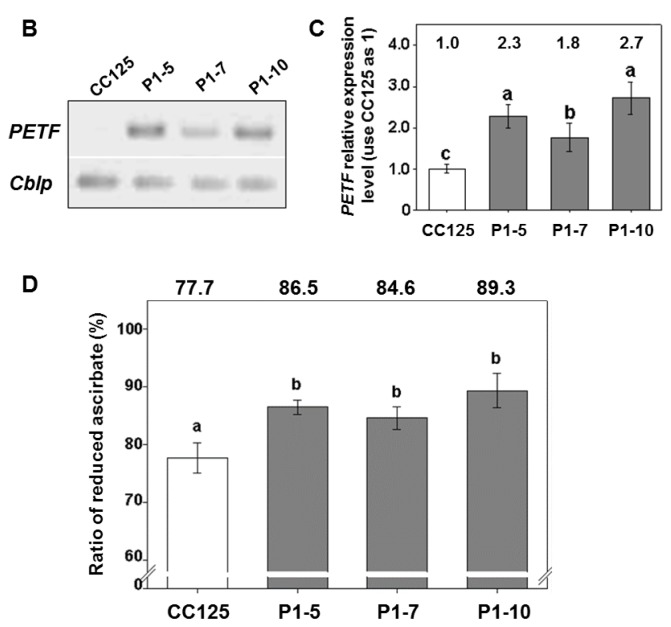
Characterizations of transgenic *Chlamydomonas* overexpressing *PETF.* (**A**) A diagram of the recombinant plasmid, pHYG3-PETF, prepared for electroporation. The coding sequence of the *PETF* gene was ligated to the sequence of β2-tubulin promoter (P_T_) to generate pHYG3-PETF. The transformants harboring the *aph7* gene can be screened using hygromycin; (**B**) To confirm putative transformants, primers 2201F and 3071R were used to amplify a part of P_T_::*PETF* and were labeled as *PETF. Cblp* was amplified as an internal control for genomic DNA; (**C**) The relative expression level of the *PETF* transcripts of algal lines under control treatment. The total mRNA transcripts obtained from foreign and endogenous *PETF* genes were quantified using primers Re-PETF-F and Re-PETF-R by qPCR. As shown above the column, the relative expression levels of the *PETF* transcripts were compared with those of the *Cblp* transcripts, and were normalized according to the value of CC125 under normal growth conditions; (**D**) Ratios of reduced ascorbate in *Chlamydomonas* cellular extract under normal growth conditions. The contents of ascorbate in the late-log phase cell cultures were measured. The ratios presented are the percentages of reduced ascorbate in total ascorbate pools of cells. Error bars indicate the standard deviation of the mean of ascorbate ratios in the cells for five repeats, and letters on the columns indicate the significant differences based on the Duncan’s multiple range test (*p* < 0.05). CC125 is a non-transformant cell line, and P1-5, P1-7 and P1-10 are *Chlamydomonas* transgenic cell lines.

PETF Using an electroporation transformation system, three transgenic *Chlamydomonas* lines, P1-5, P1-7 and P1-10, carrying a recombinant *Chlamydomonas* FDX gene, *PETF*, under the control of constitutive β2-tubulin promoter (P_T_), were generated (Figure 1A). The P_T_::*PETF* transgene was detected in all three PETF-transgenic lines by using genomic DNA PCR (Figure 1B). Furthermore, the levels of total *PETF* mRNA in all three transgenic lines were 1.7- to 2.7-fold higher than that of the non-transgenic line (CC125), as shown using quantitative RT-PCR (Figure 1C). Cellular protein extracts were probed with an antiserum of PFLP, a photosynthetic type FDX isolated from sweet pepper [36], however, no signal was detected, possibly because of its low affinity to the *Chlamydomonas* FDXs.

Ascorbate is one of the major antioxidant metabolites in plant tissues. To determine whether the ratios of reduced ascorbate were effected by ectopic expression of P_T_::*PETF*, the late-log phase cell cultures of transgenic lines were collected and subjected to measurement of ascorbate. The result showed that the ratios of reduced ascorbate in the P1-5, P1-7, and P1-10 lines were higher than that of CC125 (Figure 1D).

**Figure 2:**
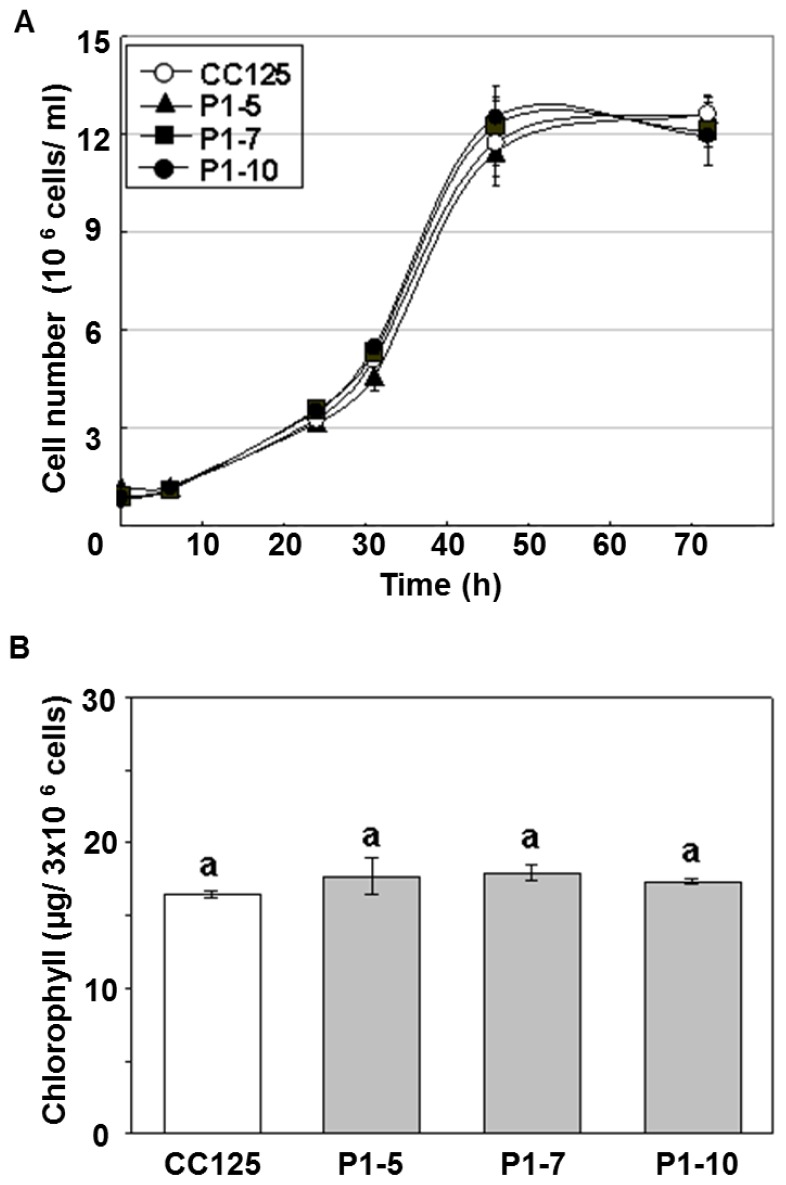
Growth curves and chlorophyll contents of transgenic *Chlamydomonas* overexpressing *PETF* under normal growth conditions. (**A**) The growth curves of non-transgenic (CC125) and *PETF*-transgenic lines (P1-5, P1-7, and P1-10) under normal growth conditions; (**B**) The chlorophyll contents of all algal lines. Error bars indicate the standard deviation of the mean for five repeats of cell suspensions, and letters on the columns indicate significant differences based on Duncan’s multiple range test (*p* < 0.05).

Chloroplasts are known to be the main targets of ROS-related damage under environmental stresses [37]. To determine whether ectopic expression of P_T_::PETF affects growth rate and chlorophyll content, the doubling time and chlorophyll content of the transgenic lines were measured. Under normal growth conditions, the doubling time was 11.5 h for CC125 *C. reinhardtii* and 12.0–12.3 h for transgenic lines (Figure 2A). The contents of chlorophyll a, chlorophyll b, and total chlorophyll were not significantly different between non-transgenic and transgenic *C. reinhardtii* under normal conditions (Figure 2B and Table 1). These results suggested that overexpression of *PETF* did not significantly change growth behaviors.

### 2.2. Overexpression of *PETF* in *Chlamydomonas* Enhances Stress Tolerance to Heat

**Figure 3:**
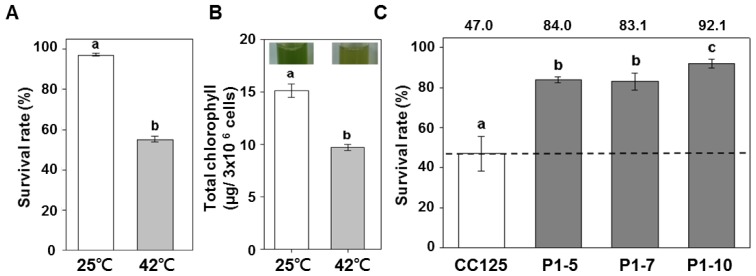
Impact of heat stress on survival rates and chlorophyll contents in *Chlamydomonas* overexpressing *PETF*. (**A**) Survival rate of *Chlamydomonas* CC125, after a 2 day recovery from heat treatment at 42 °C for 40 min; (**B**) *Chlamydomonas* cell cultures were imaged, and the total chlorophyll contents were measured immediately after heat treatment; (**C**) Survival rate comparison between non-transformant and *PETF* overexpression lines after heat treatment. The heat-treated cell cultures were incubated under normal growth conditions for 72 h prior to measuring the survival rate. Then the survival rates of the non-transgenic CC125 and transgenic lines (P1-5, P1-7, and P1-10) were measured using trypan blue staining, and the cell count of CC125 without heat treatment was determined to be 100%. Error bars indicate standard deviations of the mean for five repeats of each line, and letters indicate the significant differences based on Duncan’s multiple range test (*p* < 0.05).

Hema *et al.* reported that a growth temperature of 42 °C markedly inhibited the growth of *C. reinhardtii* cells [28]. To determine whether PETF provided protection under heat stress, we compared the survival rates of the P_T_::*PETF* transgenic lines with those of non-transformant lines under heat-stress. Cells were subjected to heat treatment at 42 °C for 40 min and recovered for 2 days. The cell viability of the non-transgenic line, CC125, dropped from 97.1% to 50% (Figure 3A). In addition, the color of the culture changed from greenish to yellowish, and the chlorophyll contents decreased immediately after heat treatment (Figure 3B and Table 1). By contrast, the survival rate of the P_T_::*PETF* transgenic lines was maintained at 85% (Figure 3C). The chlorophyll contents indicated that there are no significant difference for P_T_:*PETF* transgenic lines grown under normal and heat-stress conditions (Table 1). These results showed that over-expression of the *PETF* gene apparently increased the survival rates of *C. reinhardtii* under heat stress.

### 2.3. Accumulation of Reactive Oxygen Species (ROS) Is Reduced in Transgenic Lines Overexpressing *PETF*

**Figure 4:**
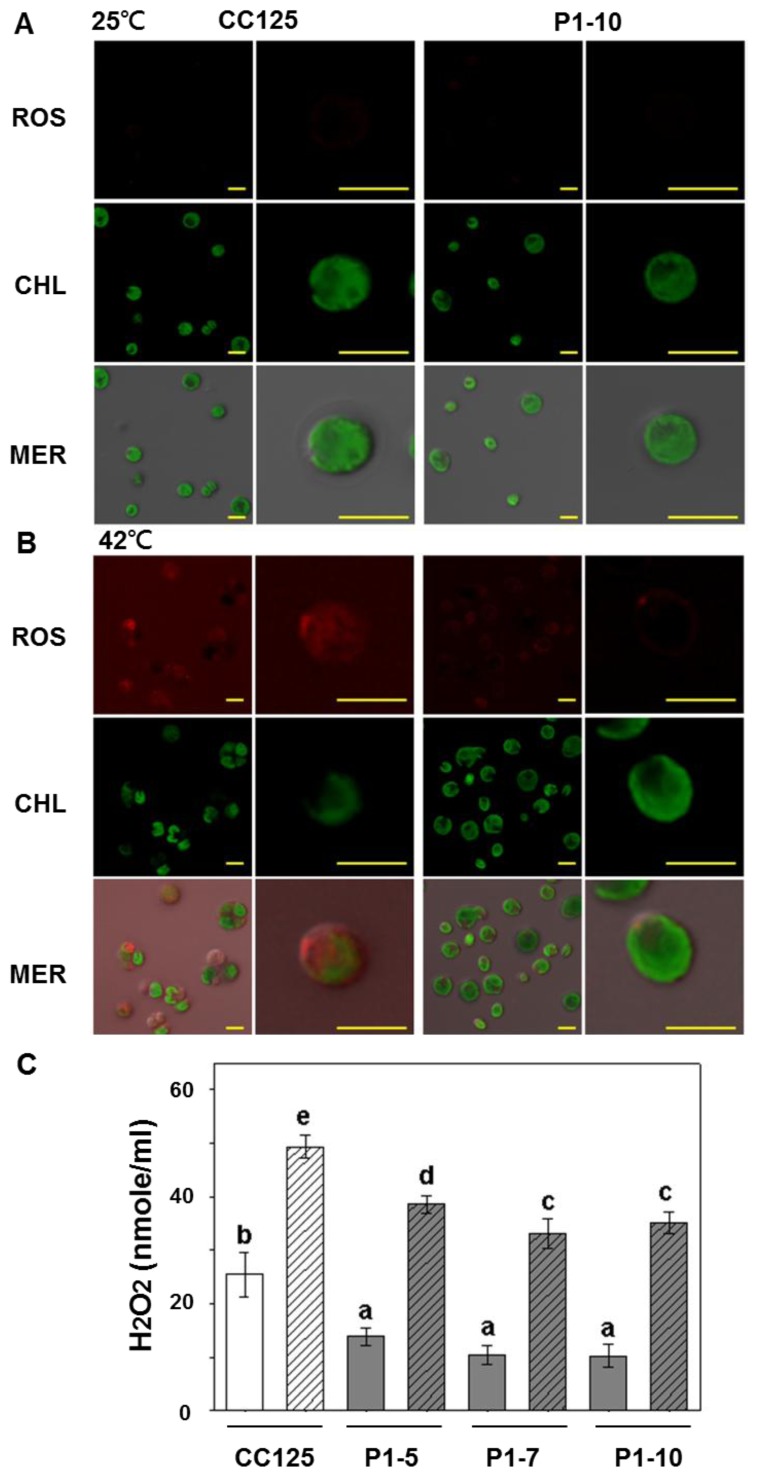
ROS and H_2_O_2_ accumulation in *Chlamydomonas* overexpressing *PETF* under heat treatment. (**A**) ROS accumulation of cells under control treatment; (**B**) ROS accumulation of cells under heat treatment. For the heat treatment, non-transgenic CC125 and transgenic P1-10 were incubated at 42 °C for 40 min and stained with H_2_DCFDA to monitor the accumulation of cellular ROS. ROS: images of red pseudocolor indicate the existence of stained ROS. CHL: images of green pseudocolor indicate the autofluorescence of chlorophyll. MER indicates the superimposed images of ROS, CHL, and differential interference contrast (DIC). Bars indicate 10 μm in length; (**C**) The H_2_O_2_ contents in the non-transgenic CC125 and transgenic lines (P1-5, P1-7, and P1-10) were measured under control (blank columns) or heat treatment (twilled columns). Cells were collected for chemical measurement immediately after heat treatment. Error bars indicate standard deviations of the mean for five repeats of cell suspensions, and letters on the columns indicate the significant differences based on Duncan’s multiple range test (*p* < 0.05).

High temperature is a type of oxidative stress that induces ROS to cause oxidative damage in plant cells [2], and FDX participates in ROS scavenging by reducing ascorbate [18]. The P_T_::*PETF* transgenic lines showed an approximately 2-fold increase in survival rates after heat treatment at 42 °C for 40 min (Figure 3C). A cell permeable fluorogenic dye, 2′,7′-dichorofluorescein diacetate (DCFDA), was used to detect ROS by confocal microscopy. The chlorophyll autofluorescence signal was used as an indicator for detecting chloroplasts. As shown in Figure 4A, no ROS signal was detected in either the non-transgenic or transgenic lines under normal growth conditions. However, after heat treatment, the level of the ROS fluorescent signal increased and chlorophyll image declined in the non-transgenic cells, whereas a low level of the ROS signal was observed, and the chlorophyll fluorescence signals remained stable in the P1-10 cells (Figure 4B). These results indicated that the increase of PETF in a cell could reduce ROS accumulation and prevent chlorophyll degradation under heat treatment. Furthermore, the content of H_2_O_2_, a major species of ROS, was measured after heat treatment. Although the amount of H_2_O_2_ increased in both CC125 and transformants after heat treatment, H_2_O_2_ concentration was significantly lower in the transgenic lines than in the heat-treated non-transgenic cells (Figure 4C). These results showed that ROS, including the major species H_2_O_2_, were significantly reduced in the *PETF*-transgenic lines, regardless of heat treatment.

### 2.4. Expression of *PETF* Correlates Positively to Thermotolerance Ability in Transgenic Lines

**Figure 5:**
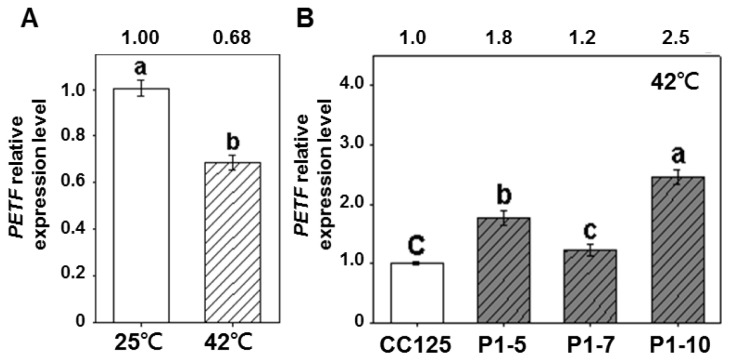
Comparison of *PETF* gene expression level under normal and heat-stress conditions. (**A**) The relative expression level of *PETF* transcripts of non-transgenic (CC125) under control (25 °C, blank columns) and heat treatment (42 °C, twilled columns); (**B**) The *PETF* gene expression level from non-transgenic (CC125) and *PETF*-transgenic lines (P1-5, P1-7 and P1-10) were specifically amplified with primers 2201F and PETF-R by qPCR. As shown above the column, the relative expression level of *PETF* transcripts were compared with those of the *Cblp* transcripts, and then were normalized according to the value of CC125 under normal growth conditions. Letters on the columns indicate the significant differences based on Duncan’s multiple range test (*p* < 0.05).

To demonstrate whether an increase in thermotolerance ability in transgenic lines is correlated to the *PETF* expression level, the mRNA quantity of *PETF* was measured using quantitative RT-PCR. After heat treatment, *PETF* mRNA in the non-transgenic lines was reduced by approximately 32% compared with normal growth conditions (Figure 5A). However, *PETF* mRNA in three transgenic lines remained at a high level after heat treatment. As shown in Figure 5B, the relative levels of *PETF* mRNA in P1-5, P1-7 and P1-10 were 177%, 123% and 245%, respectively, compared with those of the non-transgenic line (Figure 4D). The P1-10 line accumulated the highest level of *PETF* mRNA (Figure 4D) and exhibited the highest survival rate under heat treatment (Figure 3C) among the three transgenic lines. These results indicated that the levels of *PETF* transcripts were positively correlated with the thermotolerance ability in the transgenic *Chlamydomonas* lines.

## 3. Discussion

Reduction of ROS level is a major biotechnology strategy used to protect plants from various abiotic stresses [38–40]. In the study reported herein, ROS produced by *C. reinhardtii* cause oxidative damage, which results in cell death under heat treatment at 42 °C for 40 min. Three *Chlamydomonas* transgenic lines overexpressing *PETF* were generated, and ROS levels in these transgenic lines were significantly reduced even after heat treatment. These transgenic algae presented highly thermotolerant phenotypes that are correlated to the transgene *PETF* expression levels. These findings indicate that overexpression of the *PETF* gene decreases ROS levels and contributes to the tolerance of heat stress. However, plant responses to heat stress are highly complex, and have effects on protein denaturation, membrane destabilization, metabolic equilibration, and redox homeostasis [9,41]. Integration of different protective mechanisms contributes to plant tolerance under heat stress, and the complex protective networks can be facilitated by overexpression of *PETF*.

The *Chlamydomonas* genome contains six ferrdoxin (FDX) genes and the expression of each FDX gene is responsive to different environmental stress and nutrient conditions [34]. For example, transcription of *FDX2* was upregulated by H_2_O_2_, although the FDX2 protein was rapidly damaged after H_2_O_2_ treatment. On the other hand, the expression of the *FDX5* transcript was responsive to O_2_, copper, and nickel supplementation [34]. The most abundant *FDX* transcript found in *Chlamydomonas* grown in TAP medium under normal growth condition is *PETF*, and its expression remains constitutive in most tested conditions, including under H_2_O_2_ treatment [34]. In this study, it was demonstrated that *PETF* mRNA decreased slightly after heat treatment, and Terauchi *et al.* (2009) showed that PETF protein is not significantly degraded under oxidative stresses [34]. Therefore, it was proposed that high-level *PETF* mRNA can be maintained and translated to functional *PETF*, which contributes to transgenic algae resistant to heat stress. Moreover, it is known that monodehydroascorbate (MDA) is a major sink of photosynthetic electrons and can be reduced to ascorbate by FDX in cells [28,37]. Our results showed that the ratios of reduced ascorbate in the P1-5, P1-7, and P1-10 lines were higher than that of CC125 (Figure 1D), suggesting that the PETF-transgenic lines contained more functional PETF and reduced more ascorbate than the non-transgenic line did.

In this study, three transgenic lines of *Chlamydomonas* expressing the *PETF* gene were obtained. The growth curve and chlorophyll content of transgenic lines did not have significant differences compared to that of non-transgenic lines under normal growth conditions. However, after heat treatment, the survival rates of *PETF*-overexpressing lines increased significantly compared to that of non-transgenic line. In addition, chloroplasts in transgenic cells remained intact and exhibited little chlorophyll content decrease after heat treatment. Interestingly, chlorophyll b (Chl b) was more protected than chlorophyll a (Chl a) in transgenic lines. This report indicated that maintenance of chlorophylls by PETF would protect *Chlamydomonas* against heat stress. However, the mechanism of PETF-mediated chlorophylls protection needs further investigation. Plant membrane systems including thylakoid membrane are known as direct targets of ROS under heat stress [1]. Biosynthesis and degradation of chlorophyll determine the amount of chlorophyll present, and both processes are known to require FDX-dependent enzymes in plants [42–46]. Chlorophylls and their binding proteins form complexes when they are inserted into thylakoid membranes. When chlorophyll–protein complexes are dissociated, chlorophyll molecules enter the degradation pathway [47]. Therefore, there is a strong correlation between thylakoid membrane stability and chlorophyll degradation under heat conditions. Moreover, several reports showed that degree of lipid saturation in membranes increases in plants under high temperature and thus reduces membrane stability [48,49]. Saturated fatty acid containing membrane glycerolipids are converted to unsaturated fatty acid by desaturases in plastids and endoplasmic reticulum (ER) [50,51], and the plastid desaturases required FDX to provide electrons for fatty acid desaturation [42]. It is possible that overexpression of *PETF* facilitates electron donation to desaturase for desaturation of fatty acids, and hence maintains membrane stability and chlorophyll content under heat stresses.

Chl b is synthesized from and can be reconverted to Chl a. The levels of Chl b are determined by the activity of three enzyme reactions; conversion of Chl a to Chl b by chlorophyllide a oxygenase (CAO), conversion of Chl b to 7-hydroxymethyl Chl a (HMChl a) by Chl b reductase (CBR), and conversion of HMChl a to Chl a by 7-hydroxymethyl-chlorophyll reductase (HCAR) [52]. The CAO has been suggested to accept electrons from FDX to convert Chl a to Chl b, and both CBR and HCAR are FDX-dependent enzymes [42]. Overexpression of *PETF* in transgenic lines showed no alteration in levels of Chl a and Chl b, compared to the wild type, and it suggested that PETF contributes equally to both sides of conversion under normal growth condition. Chl b degradation is primarily performed via conversion of Chl a by CBR and HCAR and followed by two FDX-dependent enzymes, Pheide a oxygenase (PAO) and RCC reductase (RCCR) [42]. It is proposed that PETF favors electron donation to PAO and RCCR, and accelerates Chl a degradation; therefore Chl b was protected more than Chl a in transgenic lines under heat stresses.

Mitochondria and chloroplasts have been clearly recognized as main sources of ROS in plant cells [53]. In chloroplasts, increased levels of ROS are produced under adverse environmental conditions, such as drought, salt, high temperature and high-light, causing stress through the photosynthetic electron-transport chain (PETC) due to unsmooth electron flow [53–55]. In this study, the major ferredoxin PETF was overexpressed in expectations of reducing overproduction of high-energy electrons from PETC in *Chlamydomonas* cells under heat stress and then decreasing ROS production to prevent cell damage. Consequently, transgenic algae overexpressing *PETF* showed thermotolerance. Indeed, overexpression of chloroplast FNR, which is involved in the last step of PETC, increased tolerance to oxidative stress in tobacco [56]. In addition, ectopic expression of a prokaryotic flavodoxin, an electron carrier flavoprotein not found in plants, targeted to chloroplast in transgenic tobacco plants is shown to increase tolerance against various abiotic stress [22]. Similar hypotheses were tested in mitochondria of the mammalian cell line Cos-7 cells and results showed that ectopic expressed heterologous FNR and flavodoxin can protect Cos-7 cells against oxidative stress [57]. On the other hand, ferredoxin can transfer electrons to generate ascorbate, which is employed by ascorbate peroxidase (APX) to scavenge H_2_O_2_[18,20,58,59]. Although electrons from ferredoxin can provide an alternative sink to generate O_2_^−^ from O_2_ in the Mehler reaction, the reducing power of ferredoxin also acts for ascorbate reduction [60]. Results aforementioned indicated that transgenic *Chlamydomonas* lines overexpressing *PETF* generated more reduced ascorbate than non-transgenic algae did, and it can donate electrons in ascorbate-mediated ROS scavenging to detoxify ROS generated under heat stress in chloroplast.

## 4. Experimental Section

### 4.1. Cultivation of *Chlamydomonas reinhardtii*

*Chlamydomonas reinhardtii* Wild-type strain CC125 and *PETF*-transgenic lines were grown in TAP medium [61] under a continuous light (125 μE m^−2^ s^−1^) at 25 °C. Cell density was counted using a standard hemocytometer. The doubling time and growth curve were determined by methods described by Stern *et al.* [62].

### 4.2. Construction of Plasmids

To construct a plasmid for constitute expressing the *PETF* gene, a promoter region of β*2*-*tubulin* gene was amplified by polymerase chain reaction (PCR) with primers B2T-F: 5′-CGGGTACCGAATTVGATATCAAGCTTC-3′ (*Kpn*I site underlined) and B2T-R: 5′-GGGCCCGTTTGCGGGTTGTG-3′ (*Apa*I site underlined) from pHYG3 [63]. The amplified DNA fragment was digested with *Kpn*I and *Apa*I, and then ligated into pHYG3 to generate pHyG3-B2T. The *PETF* cDNA fragment was amplified by reverse transcription polymerase chain reaction (RT-PCR) with cPETF-F: 5′-GGGCCGGGCCCATGGCCATGGCTATGCGCTC-3′ (*Apa*I site underlined) and cPETF-R: 5′-ACCATACATATGTTAGTACAGGGCCTCCTCCTG-3′ (*Nde*I site underlined). The PCR product was digested with *Apa*I and *Nde*I, and ligated into pHYG3-B2T to generate pHYG3-PETF.

### 4.3. Gene Transformation of *Chlamydomonas reinhardtii*

Electroporation protocol of the method as described [64] was performed for cell transformation. Late log phase algal cell culture was quickly collected and washed with 10% Tween-20. Then, the cell pellet was resuspended with TAP broth containing 40 mM of sucrose. Before electroporation, cells were mixed with *Sca*I-digested linear form DNA of plasmid pHYG3-PETF. The mixture was transferred into a 4-mm gap electroporation cuvette at 10 °C. The electric pulse conditions were 2000 V/cm, 25 μF, and 500 Ω. After electroporation, cells were transferred to TAP broth medium and incubated at 25 °C for 72 h. The putative transgenic lines were screened on TAP plate with 20 μg/mL of hygromycin. All of the putative PETF-transgenic cell lines were confirmed by genomic PCR with primers 2201F (5′-CCACTTCTACACAGGCCACT-3′) and 3071R (5′-GGGCGACACGGAAATGTTG-3′).

### 4.4. Heat Treatment and Cell Survival Determination

For heat stress treatment, a single colony was incubated in TAP broth. The cell cultures with concentration of 3 × 10^6^ cell/mL were subjected to treatment at 42 °C under continuous light (125 μE m^−2^ s^−1^) for 40 min, and then recovered for an additional 72 h under continuous light at 25 °C. The cell survival rate was determined by trypan blue staining analysis [65]. The cell suspension was mixed with an equal volume of 0.4% (*w*/*v*) trypan blue solution. The numbers of unstained living cells (*N*u) and total cells (*N*t) were counted using a hemocytometer, and the survival rate was determined to be *N*u/*N*t × 100%.

### 4.5. Quantitative RT-PCR

Total RNA was isolated by using a plant total RNA kit (Viogene, Taipei, Taiwan). The cDNA was synthesized using Transcriptor First Strand cDNA Synthesis Kit (Roche, Penzberg, Germany) from 1 μg of total RNA. To measure the total *PETF* transcripts in PETF-transgenic and non-transgenic lines, quantitative PCR was performed by 7500 Fast Real-Time PCR Systems (Applied Biosystem, Foster City, CA, USA). Primers Re-PETF-F (5′-TGAGTGCCCCGCTGACACCT-3′) and Re-PETF-R (5′-GCACCAGCGCGGCAAGAGTA-3′) were designed for amplifying *PETF* transcripts. The *Cblp* gene encodes for G-protein beta subunit-like polypeptide is constitutively expressed in *C. reinhardtii* [47]. Primers Re-Cblp-F (5′-ACCTGGAGAGCAAGAGCATCGT-3′) and Re-Cblp-R (5′-TGCTGGTGATGTTGAACTCGG-3′) were designed according to its sequence (Genbank: X53574.1) for amplifying *Cblp* transcripts as an internal control.

### 4.6. Determination of Ascorbate Content

The amounts of reduced ascorbate (*A*r) and total ascorbate pool (*A*t) were measured as described with modification [66]. The total 3 × 10^6^ algal cells were collected and homogenized with a steel ball after freezing in liquid nitrogen. One milliliter of 6% trichloroacetic acid (TCA) was added to resuspend the algal extracts, which were centrifuged, and the supernatant was used for determining the ascorbate content. For determining the amount of *A*t, 100 μL of supernatant was mixed with 50 μL of 100 mM dithiothreitol and 50 μL of a 75 mM phosphate buffer (pH 7.0). The mixture was incubated at room temperature for 30 min. For determining the amount of *A*r, 100 μL of supernatant was added with 50 μL of deionized water and 50 μL of a 75 mM phosphate buffer for 30 min. The mixtures were then reacted with a reaction buffer (250 μL of 10% TCA, 200 μL of 43% H_3_PO_4_, 200 μL of 4% α-α′-bipyridyl and 100 μL of 3% FeCl_3_) at 37 °C for 1 h. The ascorbate concentrations were determined by the absorbance at 525 nm according to the standard curve made by 0.15–10 mM of ascorbate (Sigma, St. Louis, MO, USA) standards in 6% TCA. The ratio of reduced ascorbate in total ascorbate pools was calculated at (*A*r/*A*t) × 100%.

### 4.7. Reactive Oxygen Species (ROS) Detection and H_2_O_2_ Measurement

Two methods were used to quantify ROS formation. The first is semi-quantification using ROS staining [67], and the second is H_2_O_2_ quantification. For ROS staining, cells were stained with 10 μM of 2′,7′-dichlorodihydrofluorescein diacetate (H_2_DCFDA) for 20 min and then subjected to a confocal laser-scanning microscopy (LSM 510 META, Zeiss, Jena, Germany). Signals of H_2_DCFDA and autofluorescence of chlorophyll were visualized with excitation at 488 nm and emissions at 500–530 nm and 650–710 nm, respectively.

For H_2_O_2_ measurement, cells were treated with reagents of an Amplex Red Hydrogen Peroxide/Peroxidase Assay Kit (Molecular Probes/ Invitrogen, Carlsbad, CA, USA). In brief, a total of 3 × 10^6^ algal cells in log phase were collected by centrifugation, and the pellet was frozen in liquid nitrogen and ground using a steel ball. The cell debris was dissolved in a 100 μL of a 1× reaction buffer in the assay kit. The mixture was centrifuged and the supernatant was then used to measure the cellular H_2_O_2_ concentrations after incubation with horseradish peroxidase at 25 °C for 30 min. The H_2_O_2_ concentrations were determined by the standard curve developed using 0.2–1.0 μM of H_2_O_2_ standards. Data were collected with five repeats and statistically analyzed using Duncan’s multiple range test.

### 4.8. Determination of Chlorophyll Content

*Chlamydomonas* cells in log phase (total 3 × 10^6^ cells) were collected and resuspended in 1 mL of 80% acetone. After centrifugation, the chlorophyll content of the supernatant was measured according to optical absorbance at 663 nm and 645 nm by using a U-2001 spectrophotometer (Hitachi, Tokyo, Japan). The chlorophyll content was determined by the following Equations (1)–(3) [68]:

(1) $$\text{Chlorophyll }a\;(\mu \text{g}/\text{mL})=(22.9\times {\text{A}}_{645})-(4.68\times {\text{A}}_{663})$$

(2) $$\text{chlorophyll }b\;(\mu \text{g}/\text{mL})=(12.7\times {\text{A}}_{663})-(2.698\times {\text{A}}_{645})$$

(3) $$\text{total chlorophyll }(\mu \text{g}/\text{mL})=\text{chlorophyll }a+\text{chlorophyll }b=(20.2\times {\text{A}}_{645})+(8.02\times {\text{A}}_{663})$$

## 5. Conclusions

In this study, we generated transgenic Chlamydomonas overexpressing *PETF* to show that increasing FDX gene expression levels enhance the tolerance of algae to heat stress. Based on the expression levels of *PETF* transcripts, ascorbate content, H_2_O_2_ content, chlorophyll content, and survival rates, we concluded that *PETF* can enhance tolerance to heat stress in *Chlamydomonas*. These findings imply that the enhancement of crop tolerant to heat stress can be achieved by the regulation of ferredoxin.

## Figures and Tables

**Table 1:** Chlorophyll contents of transgenic *Chlamydomonas* overexpressing *PETF*.

	Chlorophyll contents (μg/3 × 10^6^ cells)					
	
	25 °C			42 °C		
	
	Chl ^a^	Chl ^b^	Total	Chl ^a^	Chl ^b^	Total
CC125	10.6 ± 0.6 ^a^	4.5 ± 0.1 ^a^	15.1 ± 0.6 ^a^	7.1 ± 0.8 ^a^	2.7 ± 0.2 ^a^	9.8 ± 1.0 ^a^
P1-5	11.3 ± 0.9 ^a^	4.1 ± 0.3 ^a^	15.4 ± 1.1 ^a^	10.5 ± 0.8 ^c^	4.4 ± 0.3 ^c^	14.9 ± 1.1 ^c^
P1-7	11.3 ± 0.7 ^a^	4.6 ± 0.3 ^a^	15.9 ± 1.0 ^a^	9.4 ± 0.1 ^b^	3.9 ± 0.1 ^b^	13.3 ± 0.2 ^b^
P1-10	11.3 ± 0.6 ^a^	4.5 ± 0.1 ^a^	15.8 ± 0.8 ^a^	11.1 ± 0.6 ^c^	4.9 ± 0.1 ^d^	15.9 ± 0.6 ^c^

Chlorophyll contents in cell cultures were measured immediately after control (25 °C) or heat treatment (42 °C) for 40 min. Values are the mean ± standard deviation for five repeats, and letters indicate the significant differences of each column based on Duncan’s multiple range test (*p* < 0.05).
